# Real-Time multifaceted artificial intelligence vs In-Person instruction in teaching surgical technical skills: a randomized controlled trial

**DOI:** 10.1038/s41598-024-65716-8

**Published:** 2024-07-02

**Authors:** Recai Yilmaz, Mohamad Bakhaidar, Ahmad Alsayegh, Nour Abou Hamdan, Ali M. Fazlollahi, Trisha Tee, Ian Langleben, Alexander Winkler-Schwartz, Denis Laroche, Carlo Santaguida, Rolando F. Del Maestro

**Affiliations:** 1 Neurosurgical Simulation and Artificial Intelligence Learning Centre, Department of Neurology and Neurosurgery, Montreal Neurological Institute, McGill University, 300 Rue Léo Pariseau, Suite 2210, Montreal, QC H2X 4B3 Canada; 2 Present Address: Faculty of Medicine and Health Sciences, McGill University, Montreal, Canada; 3 Department of Neurology and Neurosurgery, Montreal Neurological Institute and Hospital, McGill University, Montreal, QC Canada; 4 Division of Neurosurgery, Department of Surgery, Faculty of Medicine, King Abdulaziz University, Jeddah, Saudi Arabia; 5 National Research Council Canada, Boucherville, QC Canada

**Keywords:** Health care, Medical research

## Abstract

Trainees develop surgical technical skills by learning from experts who provide context for successful task completion, identify potential risks, and guide correct instrument handling. This expert-guided training faces significant limitations in objectively assessing skills in real-time and tracking learning. It is unknown whether AI systems can effectively replicate nuanced real-time feedback, risk identification, and guidance in mastering surgical technical skills that expert instructors offer. This randomized controlled trial compared real-time AI feedback to in-person expert instruction. Ninety-seven medical trainees completed a 90-min simulation training with five practice tumor resections followed by a realistic brain tumor resection. They were randomly assigned into 1-real-time AI feedback, 2-in-person expert instruction, and 3-no real-time feedback. Performance was assessed using a composite-score and Objective Structured Assessment of Technical Skills rating, rated by blinded experts. Training with real-time AI feedback (n = 33) resulted in significantly better performance outcomes compared to no real-time feedback (n = 32) and in-person instruction (n = 32), .266, [95% CI .107 .425], *p* < .001; .332, [95% CI .173 .491], *p* = .005, respectively. Learning from AI resulted in similar OSATS ratings (4.30 vs 4.11, *p* = 1) compared to in-person training with expert instruction. Intelligent systems may refine the way operating skills are taught, providing tailored, quantifiable feedback and actionable instructions in real-time.

## Introduction

The mastery of complex bimanual psychomotor surgical skills occurs in a dynamic operative room environment. This involves the continuous interplay between the learner and surgical educator focused on ongoing skills assessment and trainee personalized instruction to achieve optimal patient care and outcomes. Surgery is a high-stakes intervention where surgical instructors play crucial pedagogical roles. One of their key responsibilities is to prevent surgical errors occurring in this operative domain that may result in increased patient morbidity and economic burden^1–4^. This conventional surgical teaching model is often limited to human expert observation^5^. Consequently, it lacks standardization and objectivity and has challenges in defining, evaluating, quantifying, and teaching the composites of surgical expertise^6–8^. As a result, surgical education is implementing newer technologies into the competency-based quantifiable framework^9–11^.

The Intelligent Continuous Expertise Monitoring System (ICEMS) is a multifaceted multi-algorithm deep learning system with a rapid-decision capability for real-time applications. It is designed to mimic the role of human expert surgical instructors in the context of surgical simulation training, interacting with the learner and guiding them towards skillset mastery^12,13^. This system was integrated into the NeuroVR (CAE Healthcare) simulator—an immersive virtual reality platform for performing brain tumor resections^14,15^. The ICEMS assesses surgical performance in 0.2-s intervals and provides real-time instruction and risk detection. This system has demonstrated a granular differentiation of skill levels between experts and residents, and between residents at different stages in their neurosurgery training program^12^. Although the predictive ability of this system’s continuous performance assessment is validated, its pedagogical utility and efficiency in teaching virtually simulated surgical bimanual skills via real-time instruction and risk detection remain unexplored. In a previous study, AI-selected feedback given at the end of the procedure was more efficient than remote expert instruction^16^. However, this feedback modality did not accommodate the continuous real-time nature of surgical performance and resulted in unintended outcomes^17^. Additionally, the observed lack of improvement with remote expert instruction may not reflect the realities of intraoperative learning, where surgical educators are tasked to ensure trainee progress.

This double-blinded prospective randomized controlled trial aimed to compare the efficacy of tailored intelligent feedback provided by ICEMS to that of in-person expert instruction in simulated surgical skills training. We hypothesized that learners provided with ICEMS real-time feedback will (1) achieve a similar improvement compared to those learning in-person with expert instructors, (2) achieve a similar improvement in the Objective Structure Assessment of Technical Skills (OSATS)^5^ rating compared to those learning in-person with expert instructors, and (3) have a similar cognitive load compared to those learning in-person with expert instructors.

## Methods

This randomized controlled trial was approved by the McGill University Health Centre Research Ethics Board, Neurosciences-Psychiatry. This study was registered at the ClinicalTrials.gov, trial registration number: NCT05168150, trial registration date: 23/12/2021. This report followed the extensions of the CONSORT 2010 Statement, guidelines for the reporting of multi-arm parallel group randomized trials and interventions involving AI^18–20^.

### Participants

**Figure 1:**
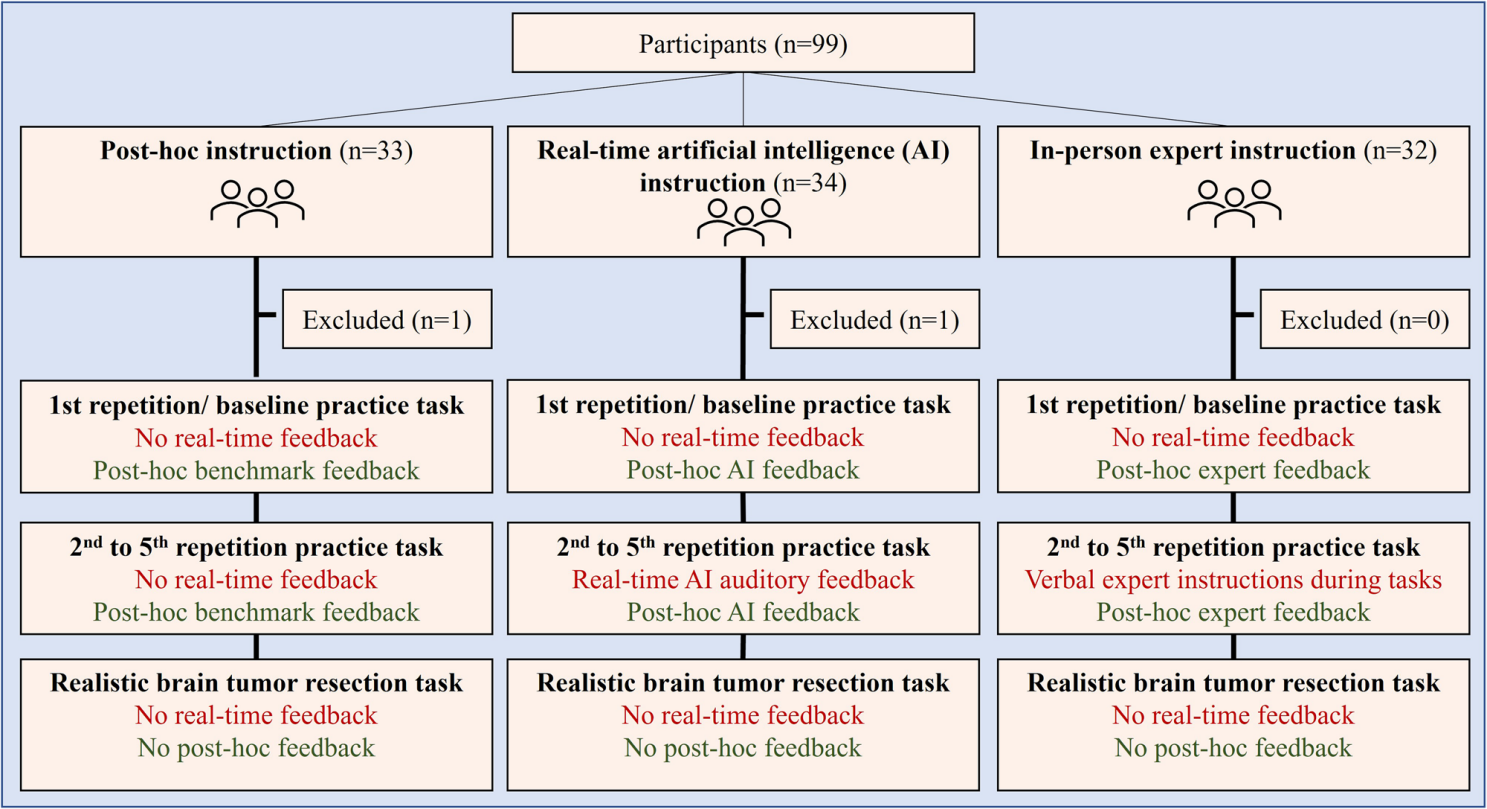
Flow diagram.

Participants were recruited between January 2022–March 2022, for a single 90-min simulation session with no follow-up (Fig. 1). The inclusion criterion was enrollment in years one to four of a medical school program in Canada. The exclusion criterion was previous experience in using the NeuroVR (CAE Healthcare). All participants signed an informed consent before the start of the trial. Public health measurements and the Montreal Neurological Institute and Hospital’s regulations related to the COVID-19 pandemic were followed to ensure health safety. Methods remained unchanged after trial commencement. The study protocol was in accordance with the ethical standards of the responsible committee on human experimentation (institutional and national) and with the Declaration of Helsinki^21^. All participants completed two questionnaires; a pre-questionnaire related to demographics, previous simulation experience and surgical exposure and, a post-questionnaire to rate their cognitive load and simulation learning experience. Participants were informed that the study involved no harm to participants and that their information is anonymized. Participants were blinded to the study outcomes.

### Randomization

Randomization into three groups was applied without stratification using an online free-access tool^22^.

### Simulation

Participants were given a standardized instruction sheet before the simulation session. The sessions were carried out in a controlled distraction-free environment. Two tumor resection tasks were performed; a practice subpial tumor resection task and a realistic brain tumor resection (Video)^15^. Expert execution of subpial technique is important in a variety of neurosurgical procedures to remove abnormal tissues while preserving the neurologic function^23,24^. The NeuroVR (CAE Healthcare, Canada) 3D neurosurgical simulation platform with two haptic handles was utilized to simulate the tasks^14^. Both tasks required using two instruments, an ultrasonic aspirator, and a bipolar forceps, to completely remove the simulated tumor while minimizing bleeding and damage to surrounding healthy tissue^25,26^. Face and content validity of the simulation tasks were previously demonstrated^15,26–28^. The time limit was five minutes for the practice task, and 13 min for the realistic tumor resection task.

Feedback was incorporated in two stages: during the task (real-time), and after the task (post hoc). Participants were randomly allocated into three groups, (1) post hoc-only feedback (active control), (2) real-time and post hoc intelligent instruction (ICEMS group), and (3) real-time and post hoc expert instruction (expert instructor group). Participants completed the practice task five times. The first repetition was completed without feedback during the performance to determine baseline. After completion of the baseline performance, participants received post hoc feedback based on their group allocation, as described in detail below. Five minutes was given for post hoc feedback for all groups. Finally, all participants performed a realistic brain tumor resection task once without feedback to assess skill transfer to this more complex simulated procedure.

### Post hoc feedback group

Participants in this group received no real-time feedback during the tasks. After the baseline and after each task, participants were provided with post hoc feedback on their performance scores in comparison to expert benchmarks on five performance metrics, which included the same metrics listed in the next section. The goal was to meet all five benchmarks by the last repetition of the task.

### Real-time AI instruction

**Figure 2:**
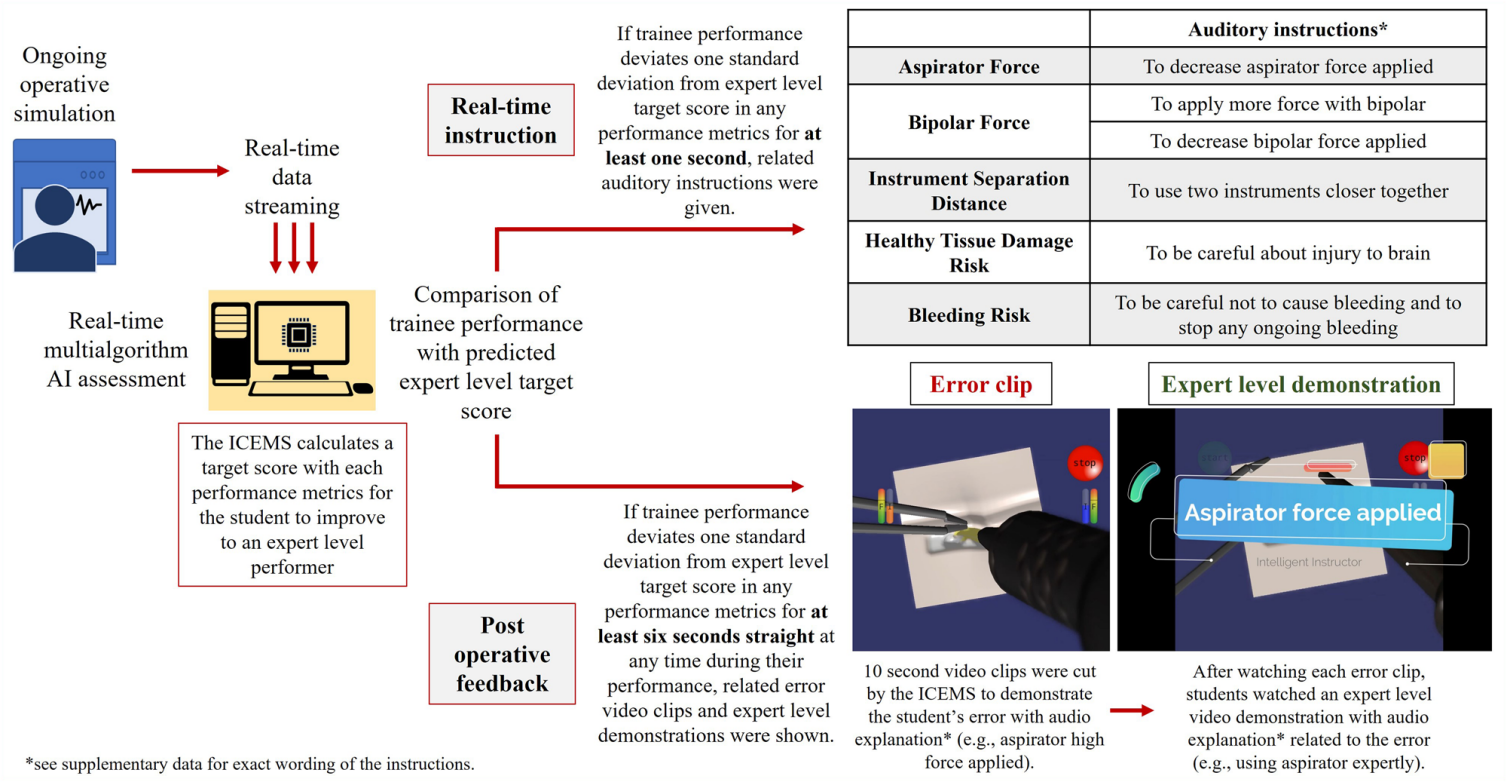
Real-time and post hoc ICEMS Feedback.

Participants in this group received real-time auditory instructions given by the ICEMS (Fig. 2)^12^. The ICEMS assessed surgical performance at 0.2-s intervals on five performance metrics: (1) bleeding risk, (2) healthy tissue damage risk, (3) ultrasonic aspirator force utilization, (4) bipolar instrument force utilization and (5) using the two instruments together. Six auditory instructions (one instruction per performance metrics and two instructions for bipolar high and low force utilization) were incorporated. ICEMS predicted expert level performance metrics in real-time based on the actions being performed by the learner. An error was identified when participant performance score differed more than one standard deviation from the expert level assessment of the ICEMS, for at least one second. Real-time auditory instructions were automatically delivered upon error identification during all practice tasks except the baseline performance. The technical background of the ICEMS and the real-time assessment and feedback was previously outlined^12,13^. The ICEMS is composed of six long-short term memory network algorithms: one for objective skill assessment and five (as listed above) for risk detection and feedback.

### Post hoc AI instruction

The participants’ performance was video recorded. After the completion of each practice task, including the baseline performance, the ICEMS located the timing of specific errors using the performance data (Fig. 2). The ICEMS cut these error footages from the entire performance video clip and demonstrated them to the participants. An error video-clip relating to each performance metrics, to a maximum total of six error video-clips were shown to the participant in the form of 10-s video-clips (see Supplementary information). An example of an expert-level video demonstration (Video-1) and a 10-s error video clip (Video-2) can be found online.

### Real-time expert instruction

Two neurosurgery residents (M.B. and A.A., post-graduate year six) provided in-person real-time instructions. To facilitate standardization, they used a modified OSATS rating scale (see Supplementary Information) and a modified PEARLS debriefing script^29^. Instructors were blinded to the ICEMS assessment metrics. These verbal instructions were provided to the students from the second repetition of the practice task to the fifth repetition during the simulated tasks.

### Post hoc expert instruction

After the completion of each practice task, including the baseline performance, the expert instructor had five minutes with the participant to outline any pertinent information to enhance performance. The expert instructors also had the option to personally demonstrate strategies and surgical techniques on the NeuroVR simulation on how to expertly perform the simulated subpial resection.

### Outcome measures

All performance data was recorded along with the video recordings of each task. The primary outcome measure was the composite performance score quantified by the ICEMS during practice and realistic tumor resections. The ICEMS scored participants’ performance between a score of − 1 (novice) and 1 (expert) at 0.2-s intervals as previously outlined^12^. An average composite-score was calculated for each repetition of the task for statistical comparisons. To outline the reasons behind differences between groups with this score, scores in five learning outcomes were analyzed, including bleeding risk, tissue injury risk, aspirator force, bipolar force, and instrument tip separation. The video recordings of the realistic brain tumor resection task were rated by two blinded expert raters using the OSATS scale as previously described^5,16^. Cognitive load was assessed through a questionnaire before, during, and after the simulation exercises^16^.

### Statistical analysis

Data was not normally distributed as assessed by Shapiro–Wilk’s test (*p* < .05). Non-parametric statistical tests were utilized. The composite score across five repetitions of the practice task was compared using Friedman’s test to examine the learning curves. Between group comparisons at each repetition of the task was done using Kruskal–Wallis H Test. The composite score on the realistic task was normally distributed as assessed by Shapiro–Wilk's test (*p* > .05). The composite score is compared by a one-way ANOVA to assess learning transfer to this more complex brain tumor resection task. The OSATS score on the same task was compared between groups by a Kruskal–Wallis H Test. Outliers were observed by visual examination of boxplots. Outliers were imputed using the nearest non-outlier value. Levene’s test showed heterogeneity of variances, based on median (*p* < .05), and Box’s test demonstrated violation of homogeneity of covariances, *p* < .001. The assumption of sphericity was violated for the two-way interaction as indicated by Mauchly's test, χ^2^(9) = 32.54, *p* < .001. Hence, the results with Greenhouse–Geisser correction are reported. Post hoc pairwise comparisons were adjusted by Bonferroni correction for multiple tests. IBM SPSS Statistics, Version 27 was used to conduct statistical analyses.

## Results

### Participants and sample size

Ninety-nine medical students who were presently enrolled in four medical schools across the province of Quebec participated in this three-parallel-arm randomized controlled trial (Fig. 1). Participant simulation performance data was recorded in one session without a follow-up. Data from two participants was excluded from the analysis due to technical issues faced during the simulated tasks. Mean participant age + /− SD (Range) was 21.3 ± 2.7 (17–31) years, and participant handedness was 89/7/1 (right-handed/left-handed/ambidextrous). Participants’ level of interest in surgery was a median (range) of 4 (1–5) (Table 1). A sample size calculation for a power of 0.9 with an effect size of 0.3, 0.5 correlation among repeated measures yielded 30 participants in each group, and 90 participants in total, for between-group comparison. Data analysis was conducted based on intention-to-treat.

**Table 1:** Participant characteristics.

	Group 1	Group 2	Group 3	All participants
	Post-hoc feedback	Real-time AI feedback	Expert instruction	
	(n = 32)	(n = 33)	(n = 32)	(n = 97)
Mean age + / − SD (range)	21.1 +/− 2.4 (19–26)	21.4 + / − 3.0 (17–31)	21.3 + / − 2.8 (17–31)	21.3 + / − 2.7 (17–31)
Male/Female	10/22	14/19	15/17	39/58
Handedness (Right/left/Ambidextrous)	28/4/0	30/3/0	31/0/1	89/7/1
Year in medical school:				
Preparatory year	9	8	9	26
1st	20	23	13	56
2nd	3	1	6	10
3rd	0	0	4	4
4th	0	1	0	1
Level of interest in surgery, median (range)	4 (2–5)	4 (2–5)	4 (1–5)	4 (1–5)
Completed surgical rotation (Y/N)	0/31	1/33	3/29	4/93
Medical School:				
McGrill University	15	16	12	43
University of Montreal	10	6	8	24
University of Sherbrooke	0	4	6	10
University of Laval	7	7	6	20
Playing video games:				
Not at all	18	24	17	59
1–5 h per week	11	6	11	28
6–10 h per week	2	1	2	5
> 10 h per week	1	2	2	5
Playing musical instruments (Y/N)	15/17	15/18	16/16	46/51
Previous activities that require hand dexterity	13/19	17/16	13/19	43/54
Previously used virtual reality simulation (Y/N)	1/31	0/33	0/32	1/96

There was a significant correlation between the ICEMS’s composite score and the average OSATS score given by two expert raters, Spearman’s correlation coefficient = .224, *p* = .028. This may indicate that these two scores may be used interchangeably for performance assessment. The correlation coefficient between the two expert raters was also significant with Spearman’s correlation coefficient = .258, *p* = .011. There was a poor agreement between the two raters, κ (Cohen’s kappa) = .02 (95% CI − .039 .121), *p* = .604. These two results together suggest that the ranking order was similar between the raters; however, their exact ratings may not match.

### Between-feedback comparison

**Figure 3:**
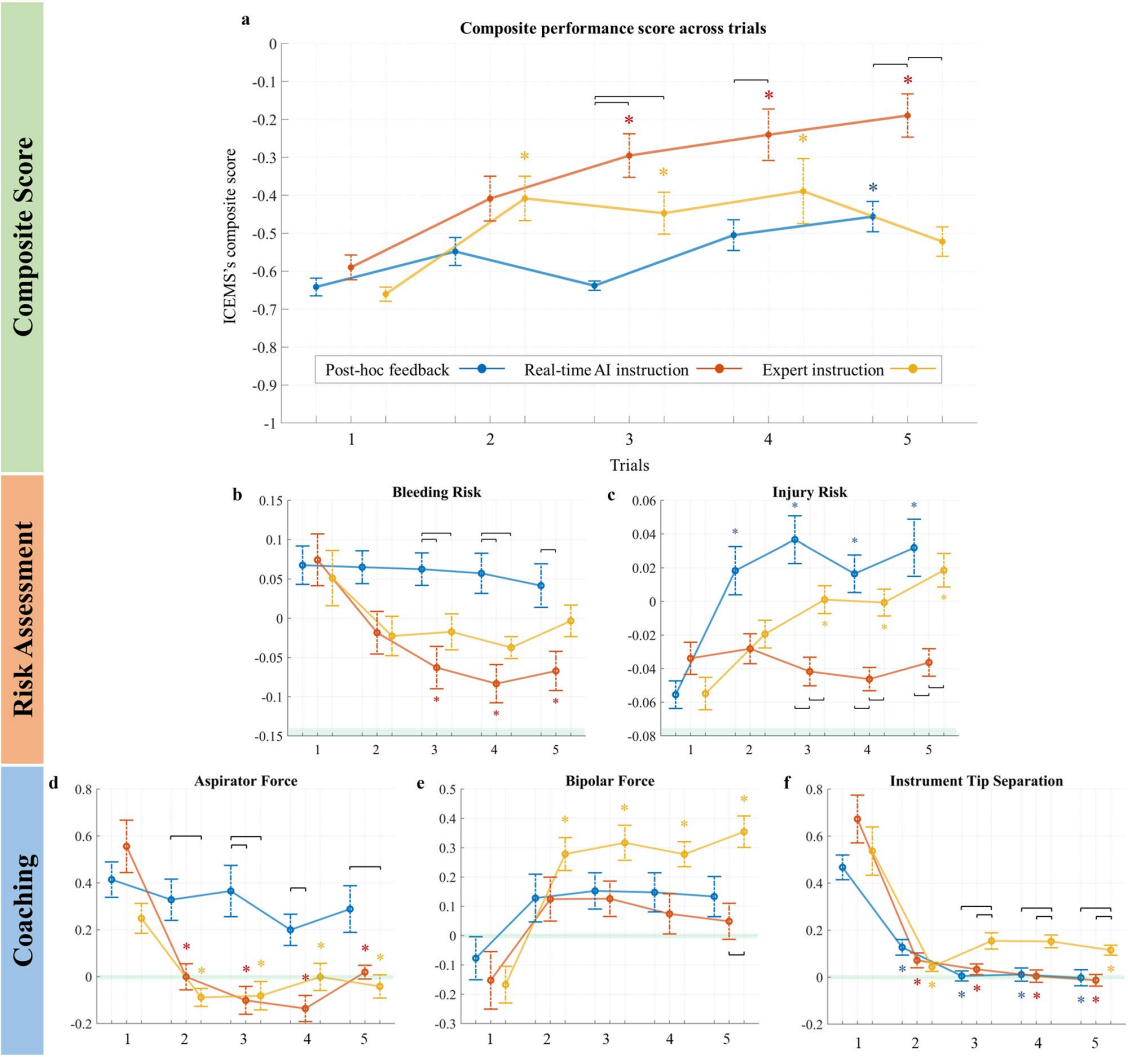
Composite score and five learning outcomes across trials. Groups are color-coded (see the legend). X-axis represents the task repetition while Y-axis represents the composite score or the scores for each of the learning outcomes. The maximum achievable composite score was + 1. *Horizontal lines represent statistically significant differences (*p* < .05). For within-group differences, these lines are shown in the respective color of the group. Vertical bars represent standard error. Colored * indicate statistically significant differences (*p* < .05) from the baseline for that group.

There were no significant differences in the composite-score in the baseline performance, *p* = .421 among the three groups (Fig. 3). There was a statistically significant interaction between feedback allocation and task repetition in a two-way mixed model ANOVA on the ICEMS composite score, *F*(6.8, 319.5) = 5.06, *p* < .001, partial η^2^ = .097. In the third task, both the ICEMS and expert instruction groups outperformed post hoc feedback group, (.343, 95% CI [.182 .504], *p* < .001), and (.190, 95% CI [.052 .330], *p* = .049), respectively. In the fifth task, the ICEMS group outperformed both post hoc and expert instruction groups, .266, 95% CI [.107 .425], *p* < .001 and .332, 95% CI [.173 .491], *p* = .005, respectively.

### Within-group learning curves

The post hoc-only feedback group improved their performance in the fifth task compared to the baseline (185, 95% CI [.039 .332], *p* = .009) (Fig. 3). The ICEMS group outperformed their baseline in the third, fourth, and fifth tasks; .295, 95% CI [.073 .516], *p* = .031, .350, 95% CI [.107 .593], *p* = .001, and .400, 95% CI [.180 .620], *p* < .001, respectively. The expert instruction group achieved a steep performance improvement in the composite-score where they outperformed their baseline performance in the second, third, and fourth tasks; .252, 95% CI [.070 .434], *p* = .001, .213, 95% CI [.054 .372], *p* = .027, .235, 95% CI [.051 .418], *p* = .016, after which they reached a plateau. There was a decrease in the composite-score and no significant difference was found between the fifth task and the baseline, .138, 95% CI [.023 .253], *p* = .269.

### Five learning outcomes

Regarding the bleeding and tissue injury risk scores (Fig. 3b and c), a lower score indicated better performance. The ICEMS group achieved significantly lower bleeding risk score by the third repetition of the task when compared to the baseline (.135, 95% CI [.021 .249], *p* = .013) while the other two groups had no statistically significant improvement. The ICEMS group achieved significantly lower tissue injury risk score in the third repetition of the task and onwards when compared to the post-hoc feedback and in-person expert instruction group − .078, 95% CI [− .113 − .042], *p* < .001 and − .041, 95% CI [− 077. − .007], *p* = .009, respectively. Students receiving in-person expert instruction caused significantly higher tissue injury risk by the third repetition of the task compared to their baseline, − .056, 95% CI [− .093 − .017], *p* < .001. For instrument utilization metrics in Fig. 3d, e, and f, a value of ‘0’ indicated no difference from the expert level. Students who receive real-time AI feedback applied significantly less bipolar force and they used their instruments closer in the fifth repetition of the task when compared to the expert instruction group − .299, 95% CI [− .503 − .095], *p* = .001 and − .143, 95% CI [− .229 − .059], *p* < .001, respectively. Students receiving in-person expert instruction applied significantly higher bipolar force by the second repetition of the task compared to their baseline, − .446, 95% CI [− .681 − .210], *p* = .004, deviating from expert-level values.

### Performance on the realistic task

**Figure 4:**
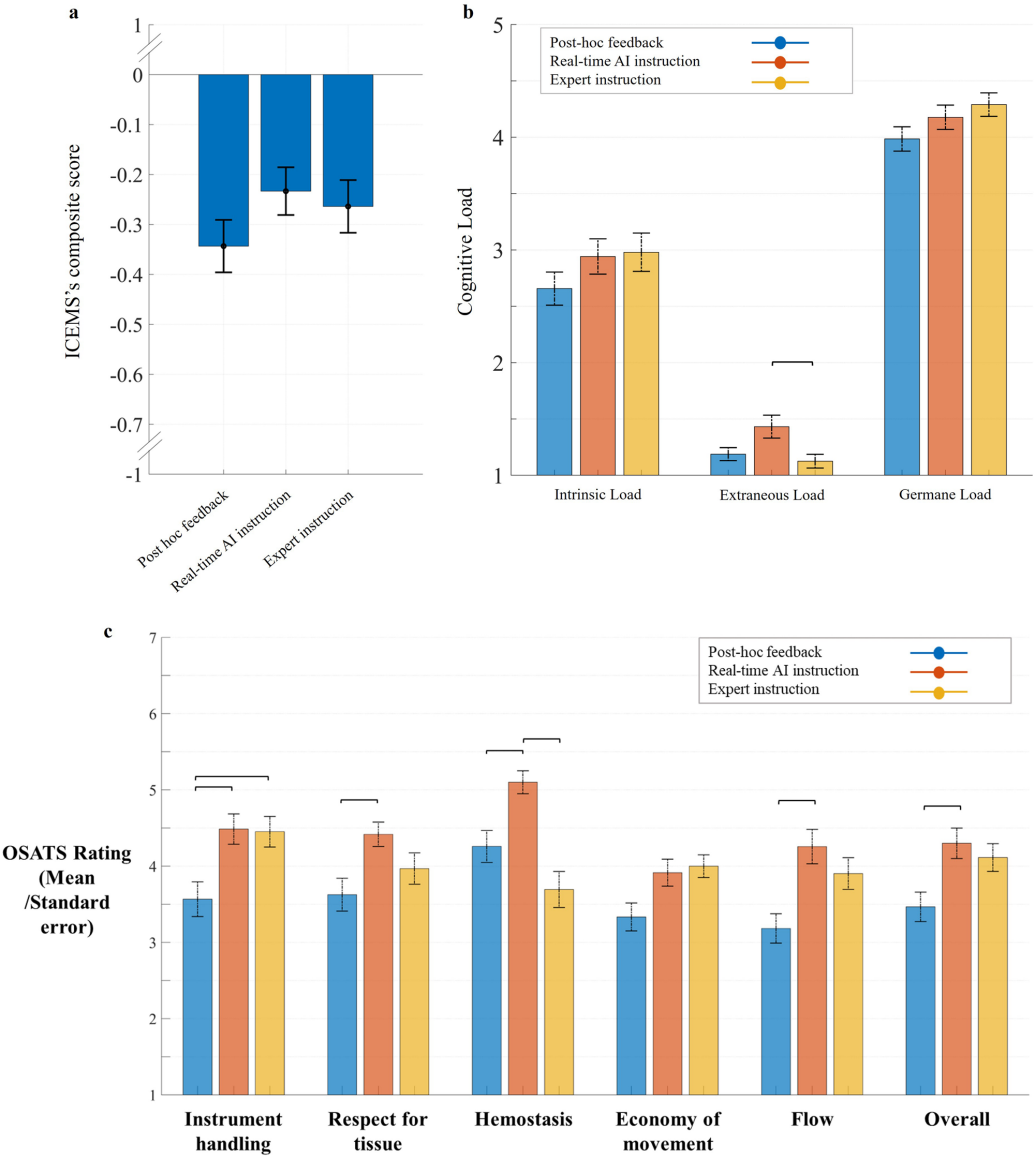
(**a**) ICEMS’s composite-score in realistic task. The vertical bars represent standard errors. There was no significant difference between three feedback groups. (**b**) Cognitive load. Groups are color-coded (see the legend). The vertical bars represent standard errors. Participants who received real-time AI instruction reported significantly higher extraneous load than those received in-person expert instruction. There were no significant differences between groups concerning intrinsic load and germane load. (**c**) Blinded expert OSATS rating. Horizontal lines represent statistically significant differences (*p* < .05). Vertical bars represent standard error.

The mean [95% CI] composite scores on the realistic task were − .343 [− .450 − .236] for post hoc feedback group, − .233 [− .330 − .136] for real-time AI group, and − .263 [− .371 − .156] for expert instruction group (Fig. 4a). No statistically significant between groups differences were observed, *F*(2, 94) = 1.241, *p* = .294.

### Blinded expert OSATS rating

The OSATS rating (median score on a 7-point scale) of the realistic task involved five items and an overall score given by two blinded experts (Fig. 4c). An average of the ratings by two experts were calculated for each item. Participants in the ICEMS group (4.30) achieved a significantly higher overall score than those in post hoc feedback group (3.47), *p* = .017. The overall score achieved by the participants in the expert instruction group (4.11) was not significantly different than both post hoc and the ICEMS groups, *p* = .137, and *p* = 1, respectively. The ICEMS group (4.9) outperformed both post hoc (4.15) and expert instruction groups (3.69) in hemostasis, *p* = .017, and *p* < .001, respectively. The ICEMS group outperformed the post hoc feedback group in instrument handling (4.49 vs 3.57, *p* = .006), respect for tissue (4.26 vs 3.73, *p* = .015), and flow (4.26 vs 3.18, *p* = .002) while the expert instruction group outperformed the post hoc feedback group only in instrument handling (4.45 vs 3.57, *p* = .014). Overall, the ICEMS group achieved better learning outcomes concerning hemostasis, respect for tissue, flow, and overall OSATS score.

### Cognitive load assessment

Intrinsic, extraneous, and germane load (median score on a 5-point scale) were assessed through the Cognitive Load Index for cognitive demands on a 5-point Likert scale (Fig. 4b)^30^. No significant differences were observed between groups in intrinsic and germane load; χ^2^(2) = 1.983, *p* = .371, and χ^2^(2) = 3.732, *p* = .155, respectively. Participants in ICEMS group (1.19) reported significantly higher extraneous load than those in expert-instruction group (1.13), *p* = .005, indicating increased cognitive difficulty experienced by the trainees in understanding ICEMS’s instructions.

## Discussion

To the best of our knowledge, this is the first randomized controlled trial that compares real-time intelligent instruction with in-person human expert instruction in teaching bimanual surgical skills in simulation training^31,32^. Our findings demonstrate superior learning outcomes using a real-time intelligent system compared to in-person expert instruction. These results were confirmed when measured quantitively by the ICEMS and when assessed by blinded experts using OSATS ratings.

The significant correlation between the ICEMS score and the blinded-expert OSATS rating may suggest that the ICEMS’s ratings may be equivalent to those of human rater. The use of ICEMS as a standalone score by possibly replacing the OSATS in simulated performance assessment needs further exploration.

Previous simulation training methodologies typically involve repetitive practice of basic to complex tasks, often without feedback or with post hoc performance feedback^16,33–37^. In both intervention arms of this study, we aimed to replicate the real-time training engagement happening in the operating room where trainees receive ongoing assessment and instructions from expert surgeons.

Feedback is critical for skill acquisition, and the most effective modalities may depend on the surgical procedure being taught^38–40^. In training for complex procedures such as the subpial resection of brain tumors, practice without feedback has resulted in little to no improvement while post hoc feedback based on performance metrics benchmarks has resulted in significant improvement in learning^16,41^. Hence, our study utilized an active control group that received post hoc feedback.

Cognitive load is the mental exertion of a trainee to process and retain information^42,43^. In this trial, learning from the real-time intelligent instructions resulted in significantly higher extraneous load, suggesting increased cognitive demand experienced by the trainees to understand the real-time auditory instructions and the post hoc video demonstrations. However, extraneous cognitive load should be minimized for more efficient learning^44,45^. This study did not assess other relevant measures related to students' learning, such as EEG, functional near-infrared spectroscopy (fNIRS), and skin conductivity. Future studies may use these measures to monitor students' cognitive load, neuronal activity, and associated stress patterns.

In this study, expert instructors had greater flexibility in their teaching engagement with students. Experts were able to provide learners with more context concerning the surgical procedure, share relevant strategies, and help students develop a plan for using the instruments to remove the tumor efficiently. They also had the option to personally demonstrate how to improve instrument performance, that mimics a technique frequently utilized by educators in the operating room. The ICEMS provided direct instructions on five performance metrics. Despite the limitations of the ICEMS, the data-driven tailored approach provided more or similarly efficient training. With the advancing techniques in AI and integration of large language models,^46^ user engagement of intelligence systems may improve substantially.

In-person expert instruction resulted in less favorable learning outcomes such as the use of too high bipolar force and an increased risk of tissue injury. This may be due to several factors such as limited human attention and judgment. First, the instructors may have difficulties in accurately quantitating many critical metrics utilized by the ICEMS including the amount of blood loss, instrument velocity, and acceleration along with the distance between instruments. This restricts their ability to provide a comprehensive assessment of trainee performance. Human instructors had limited information about how much force was applied to the tissues by the students; therefore, they were not able to address the issue of excessive force and the consequently increased risk of tissue injury^47^. Second, human attention may be limited; therefore, when their attention was on the dominant hand instrument use, they may have missed the suboptimal utilization of the bipolar forceps in the non-dominant hand, resulting in excessive force utilization. On the other hand, the ICEMS continuously monitored information regarding both instruments, tissues, and bleeding. Third, instructors may get tired, and their attention span may drop over time. This may explain the reasons behind the drop in students’ performance in the fifth repetition of the task and no significant differences from their baseline. Although expert consultation was important in the development of the ICEMS, the real-time AI capabilities may surpass the limitations of human judgment and attention. Our findings suggest that continuous AI intervention in surgical technical acquisition may be necessary to provide quality assurance and optimize learning.

The training protocol in this study was limited to a single session with no follow-up. Trainees instructed by the ICEMS system achieved a mean composite score of − 0.2 in the fifth repetition of the task, indicating that there is still significant room for improvement. Longitudinal training with multiple training sessions may be needed to improve performance further.

Although this study was conducted in a simulation training setting, the applications of intelligent instruction and assistance may not be limited to simulation settings. Methodologies are being developed to accurately identify surgical steps, potentially assess intraoperative performance during surgery, and provide feedback using artificial intelligence^48,49^. Obtaining performance data during surgery in realistic operating settings using real surgical instruments may enable transitioning intelligent feedback systems to the real operating room to mitigate errors during surgery^50–52^. Currently, computer vision systems are being implemented in the real-operating room to track information similar to the performance metrics that the ICEMS uses to make its decisions. Operative cameras are already being used in the operating room, providing great feasibility to computer vision systems without interfering with surgery and the sterile environment.

Our results have shown that in the realistic trial, the ICEMS group achieved a higher mean composite score, although no significant differences between groups were observed. This may be explained by several interacting factors: First, this challenging task may have required a greater range of skills, which caused a greater variation among students, necessitating a larger sample size to detect significant differences. Second, this difficult task may require more repetitions, similar to the practice task repetitions, for students to demonstrate their improvement and skill transfer. Third, and less likely, the disparity between the two simulated tasks assessed is such that improvement in the practice task does not result in learning transfer to the complicated realistic procedure.

Regarding the personalized feedback, the current version of the ICEMS was designed for learners with limited knowledge of brain tumor surgery. On the other hand, the ICEMS background algorithms were trained using data from neurosurgeons predominantly involved in surgical oncology and can theoretically be used to train more advanced trainees. This can be achieved by modifying the feedback scripts and video feedback of the ICEMS.A limitation of the ICEMS system is that continuous task assessment may not accurately reflect the procedural outcome^17^. In some cases, trainees may demonstrate correct instrument utilization techniques without removing sufficient tumor. Both ICEMS and OSATS assessments are more focused on instrument handling than the operative outcomes. Mixed systems may be needed to assess the expert-level procedural outcomes achieved while using correct instrument techniques. The ICEMS currently uses six algorithms to evaluate surgical performance and provide feedback in real-time^12,13^. Future versions of this system may incorporate additional modules to evaluate the procedural progress, outcome, and spatial information^27,53^.

The trainees’ skillset may affect learning and capacity for performance improvement. Our study involved medical students with little to no surgical exposure. Their limited procedural knowledge may have provided greater room for improvement in tumor resection skill acquisition, closely mirroring the situation of a novice surgical trainee at the outset of their training.

In summary, this randomized controlled trial demonstrated an effective use of a real-time intelligent system in teaching bimanual surgical tumor resection skills that is more efficient when compared to in-person instruction from human experts. Using data-driven performance monitoring and intelligent feedback may not only help to meet the needs of competency-based surgical training but also provide an effective tool to sustain technical mastery.

### Supplementary Information

Supplementary Information 1.

Supplementary Video 1.

Supplementary Video 2.

## Data Availability

The dataset analyzed in this study is available from the corresponding author on a reasonable request. A sample raw simulation data file is available online^54^.
