# Supplementary material for: Real-Time multifaceted artificial intelligence vs In-Person instruction in teaching surgical technical skills: a randomized controlled trial
Source: Sci Rep. 2024 Jul 2;14:15130. doi: 10.1038/s41598-024-65716-8 (PMC11219907; doi:10.1038/s41598-024-65716-8)
Supplement: Supplementary file 1 — Supplementary Information 1. [file 41598_2024_65716_MOESM1_ESM.docx]

**Supplementary Information**

**Real-Time Multifaceted Artificial Intelligence vs In-Person Instruction in Teaching Surgical Technical Skills – A Randomized Controlled Trial**

Recai Yilmaz, MD, PhD^1,2^*, Mohamad Bakhaidar, MBBS, MSc^1,3,4^, Ahmad Alsayegh, MBBS, MSc^1,3,4^, Nour Abou Hamdan, MSc^1,2^, Ali M. Fazlollahi, MSc^1,2^, Trisha Tee, BSc^1,2^, Ian Langleben^1,2^, Alexander Winkler-Schwartz, MD, PhD^1,3^, Denis Laroche, MSc^5^, Carlo Santaguida, MD^2,3^, Rolando Del Maestro, MD, PhD^1,2,3^

^1^ Neurosurgical Simulation and Artificial Intelligence Learning Centre, Department of Neurology & Neurosurgery, Montreal Neurological Institute, McGill University, 300 Rue Léo Pariseau, Suite 2210, H2X 4B3, Montreal, Quebec, Canada.

^2^ Faculty of Medicine and Health Sciences, McGill University, Montreal, Canada

^3^ Department of Neurology and Neurosurgery, Montreal Neurological Institute and Hospital, McGill University, Montreal, Quebec, Canada.

^4^ Division of Neurosurgery, Department of Surgery, Faculty of Medicine, King Abdulaziz University, Jeddah, Saudi Arabia.

^5^ National Research Council Canada, Boucherville, Quebec, Canada

***Standardized Instructor Training* Participant ID:**

**Performance Assessment Sheet**

Initials (rater): Date:

Subpial Scenario: Practice / Realistic Task Number:

NOTES (Things that the participant was well, the areas of improvement, instructions/feedback):

**OSATS Visual Rating – 7-point Likert Scale**

***Instrument Handling***: How would you rate this participant’s ability to handle instruments appropriately and make fluid movements?

Novice 1  2  3  4  5  6  7  Expert

***Respect for Tissue***: What is the level of care this participant shows for the tissue and the surrounding brain?

Novice 1  2  3  4  5  6  7  Expert

***Hemostasis***: How would you rate this participant’s ability to control bleeding?

*If there was no active bleeding, please check N/A:

Novice 1  2  3  4  5  6  7  Expert

***Economy of Movement***: How would you rate this participant’s efficiency of movement?

Novice 1  2  3  4  5  6  7  Expert

***Flow***: How would you rate this participant’s flow of movement in the operation?

Novice 1  2  3  4  5  6  7  Expert

***Overall***: How would you rate this participant’s overall performance in removing a considerable amount of the tumor competently?

Novice 1  2  3  4  5  6  7  Expert

**The ICEMS real-time voice instructions:**

The ICEMS made action-oriented intelligent assessment on five performance metrics to provide instruction. This system predicted values at these five-performance metrics, five predictions per second on what would be ideal for expert level. Real-time auditory feedback was given when an error was identified, when participant score on a performance metric was at least one standard deviation (a z-score difference of +1 or -1) away from the ICEMS’s expert level prediction for at least one second. For example, if force applied by bipolar instrument was too high for one second, the participant was instructed by the ICEMS to decrease bipolar instrument force applied. When an error was identified one of the voice instructions associated with that error was played randomly. If multiple errors occurred simultaneously, the order of hierarchy in the list below was followed to determine the priority of the errors. The error listed higher in the hierarchy took precedence, and an auditory feedback was provided to the student. After each auditory instruction, no further instructions were given for at least 15 seconds, allowing the students time to understand the instruction and respond.

If high tissue injury risk:

1. Please be careful and try to not damage the white healthy tissue.
2. Try to avoid damaging the healthy brain surrounding the tumor.
3. Be more cautious to not damage the surrounding healthy tissue.
4. Try to avoid any injury to the brain.

If high bleeding risk:

1. Be careful not to cause bleeding and please stop any ongoing bleeding.
2. Careful control of bleeding will improve your performance.
3. There is higher bleeding risk. To improve your performance, try to be careful not to start any bleeding.

If instruments are too separate:

1. Try to use the two instruments close together.
2. You can improve your performance by keeping the instruments closer together.
3. You can improve your performance by using the instruments together.
4. Keeping your instruments closer together will improve your performance.

If bipolar force applied is too high:

1. Try to decrease the amount of force you are applying with your bipolar.
2. Your bipolar force is too high. You can improve your performance by decreasing the force applied with your bipolar.

If bipolar force applied is too low:

1. You can apply more force with your bipolar. (If the student applies force but not enough).
2. You can improve your performance by applying more force with your bipolar. (If the student applies force but not enough).
3. Use your bipolar more efficiently (If the student doesn’t apply force).
4. Use your bipolar to assist your dominant hand. (If the student doesn’t apply force).

If aspirator force applied is too high:

1. Try to decrease the amount of force you are applying with your aspirator.
2. Your aspirator force is too high. You can improve your performance by decreasing the force applied with your aspirator.

**The ICEMS post hoc feedback:**

**User Interface.** When an error is identified, related button is activated and become accessible to the student for them to click and watch the error they made. Expert level demonstration videos (buttons below for each error) were always available for the student to watch whenever they feel necessary. With each of the clips, the participant was provided with an expert level performance video, relevant to the error identified, to demonstrate how to expertly perform and avoid the same mistake in the next repetitions. The participants had 5 minutes to watch all the videos, and they were allowed to start from any video they considered critical. They proceeded to the next task repetition when the time limit was reached, even if they had not been able to complete reviewing all the video instructions.


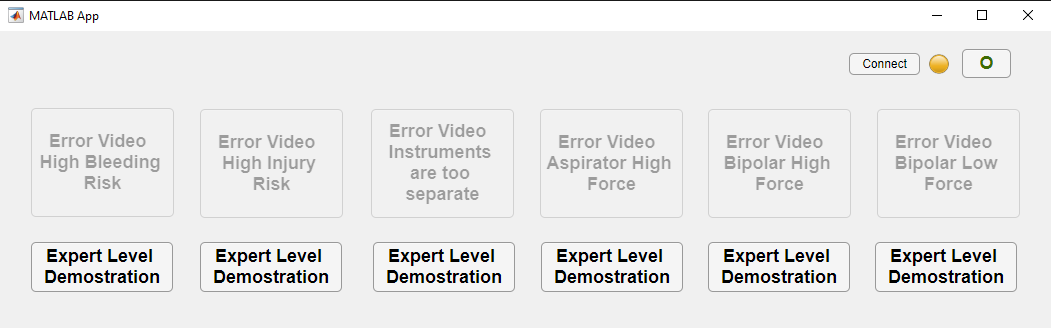


**Video background voice instructions.**

**High Bleeding Risk**

Error Video voice background (duration: 10 seconds):

In this part of the procedure, a high bleeding risk was detected. Note that there may or may not be bleeding going at this time, however the way the instruments were used during this period was associated with high risk of bleeding. Please check the expert level demonstration to learn about how to minimize bleeding risk.

Expert Level Demonstration voice background (duration: 40 seconds):

The amount of bleeding is an important safety measure in tumor surgery. To avoid bleeding, try to always use your bipolar to allow better visualization of the area you are interacting with. Be cautious when removing tumor close to the white healthy area. If bleeding occurs, stop the bleeding using your bipolar by keeping the tips of the instrument close together, but not touching. Press the pedal to activate the bipolar, which will cause an electric current to pass between the tips. Stop the bleeding by placing the bleeding point appropriately between the bipolar tips. You can use the suction capability of aspirator to remove the blood that blocks your view, without activating its pedal.

**High Injury Risk**

Error Video voice background (duration: 10 seconds):

During these moments high tissue injury risk was detected. The brain is a delicate organ. Damaging brain may result in serious consequences to patient’s function. Please check the expert level demonstration to learn how to minimize brain injury risk.

Expert Level Demonstration voice background (duration: 30 seconds):

In this simulated task, the white area demonstrates the surrounding healthy brain tissue. Attention needs to be given to not damage this white area and the blood vessel above the yellow simulated tumor. Try to be careful when you are removing tumor areas close to the tumor-brain interface (edge). Make sure to use bipolar to lift the overlying tissues and increase the visibility of the area you are removing. Stay focused, and don’t apply too much force with your both instruments, especially with your aspirator while the pedal is pressed.

**Instruments Are Too Separate**

Error Video voice background (duration: 10 seconds):

At this period, your instruments were too far apart. Not using the two instrument tips close together decreases your overall efficiency and increases the possibility of tissue injury. Please check expert level demonstration to learn how to use two instruments closely together properly.

Expert Level Demonstration voice background (duration: 20 seconds):

Using both instruments together is critical to complete the tumor resection safely and efficiently. Make sure that you always use the two instruments in proximity to improve your precision within the target area you would like to remove. Pia mater overlays brain and tumor. With your non-dominant hand, lift the pia mater off the tumor gently and use aspirator in your dominant hand to remove the underlying tumor.

**Aspirator High Force**

Error Video voice background (duration: 10 seconds):

In this part of the procedure, you applied too much force with your aspirator. High aspirator force application can result from improper handling of this instrument, and this may cause tissue injury. Please check the expert level demonstration to see how to improve your ability to use the aspirator properly.

Expert Level Demonstration voice background (duration: 40 seconds):

The aspirator is the main tool in this simulated operation which allows you to remove the tumor by pressing the pedal. You can also use this instrument to suction the blood. Remember that you don’t need to press the pedal for suctioning. Try to stay focused and gently use this instrument to avoid tissue injury. One method to achieve lower forces, is to lessen side contacts with the aspirator on surrounding tissues by adjusting the angle, by using the instrument more vertically while in contact with the tissue. You should always use bipolar to support your aspirator to help increase the visibility of the area you are interacting with. Finally, make sure that your both arms are well supported.

**Bipolar High Force**

Error Video voice background (duration: 10 seconds):

In this part of the procedure, high force application by bipolar has been detected. Excessive force application can damage the brain therefore controlled force application with bipolar is critical. Please check the expert level demonstration to see how to use the bipolar properly.

Expert Level Demonstration voice background (duration: 20 seconds):

Bipolar allows you to manipulate the tissues and expose tumor areas you would like to remove. Use bipolar to lift the tissue close to tumor edge. Gently rotate your instrument and utilize minimal force necessary to expose the tissues beneath. Stay focused. And finally, keep your arms comfortable and well supported.

**Bipolar Low Force**

Error Video voice background (duration: 10 seconds):

In this part of the procedure, force applied by bipolar was lower than required for good outcome. Not using bipolar properly may decrease the efficiency in your performance. Please check the expert level demonstration to learn how to efficiently use bipolar.

Expert Level Demonstration voice background (duration: 20 seconds):

Efficiently using bipolar allows you to better expose tumor areas and safely remove tumor with the aspirator. While removing tumor, make sure to consistently use bipolar always to assist your dominant hand, by lifting the tissues above the tumor area you would like to remove. Make gentle movements to help to better expose the underlying tissues.
